# Evaluation of a Cervical Cancer Screening Program Based on HPV Testing and LLETZ Excision in a Low Resource Setting

**DOI:** 10.1371/journal.pone.0013266

**Published:** 2010-10-07

**Authors:** Margaret McAdam, Jerol Sakita, Len Tarivonda, James Pang, Ian H. Frazer

**Affiliations:** 1 The University of Queensland Diamantina Institute, The University of Queensland, Brisbane, Queensland, Australia; 2 Department of Public Health, Ministry of Health, Port Vila, Efate, Vanuatu; Karolinska Institutet, Sweden

## Abstract

We conducted studies in Vanuatu to evaluate potential screening and treatment strategies to assist with control of cervical cancer. In a pilot study of 496 women, visual inspection and cytology were evaluated as screening tests for detection of CIN 2 or worse (CIN2+), observed in 21 of 206 subjects biopsied on the basis of abnormal visual inspection or cytology. Sensitivity of visual inspection with Lugol's Iodine for detection of CIN2+ on biopsy was 0.63, specificity was 0.32, and the positive predictive value was 0.09. For HSIL cytology, sensitivity was 0.99, specificity was 0.77, and the positive predictive value was 0.88. HSIL cytology was significantly more sensitive and had a significantly higher PPV for CIN 2+ than visual inspection (p<0.01). In a further study of 514 women, we compared testing for HR HPV and cytology as predictors of biopsy proven CIN 2+. Sensitivity of HSIL cytology for CIN2+ as established by loop excision of the cervix was 0.81, specificity was 0.94, and positive predictive value was 0.48. Sensitivity of a positive test for HR HPV for detection of CIN2+ was non-significantly different from cytology at 0.81, specificity was 0.94, and positive predictive value was 0.42. Combining the two tests gave a significantly lower sensitivity of 0.63, a specificity of 0.98, and a positive predictive value of 0.68. For women over 30 in a low resource setting without access to cytology, a single locally conduced test for high risk HPV with effective intervention could reduce cervical cancer risk as effectively as intervention based on cytology conducted in an accredited laboratory.

## Introduction

Cervical cancer is a disease of the developing world, and is responsible for over 250,000 deaths annually. Cytological screening programs can be effective for prevention of cervical cancer in the developed world, but rely heavily on effective cytopathology services and call/recall systems to ensure compliance with regular screening assessments amongst at risk women [1]. Neither of these criteria can currently be met in resource poor settings [2].

To overcome this problem, alternate strategies have been proposed to detect women in low resource settings requiring intervention to prevent cervical cancer. These include visual inspection of the cervix [3] by trained nurse practitioners, with staining enhanced by Lugols Iodine (VILI) or Acetic Acid (VIA) [4] and, more recently, testing for infection of the genital tract with high risk human papillomaviruses(HR HPV) [5], as persisting infection of the cervix with these viruses is responsible for over 98% of cervical cancer. There have been varying reports of the efficacy of these assays for detection of lesions requiring intervention [6]–[8], and there has also been debate about what should comprise definitive treatment for cervical cancer prevention in a low resource setting [9].

Vanuatu is a Pacific island nation with limited health care facilities and a poorly documented incidence of cervical cancer, believed similar to the high rates reported for the neighbouring country of Fiji [10]. At the time of study there were no measures in place to prevent cervical cancer and minimal facility to treat disease if detected. As part of a government supported program to investigate approaches to prevention of cervical cancer through vaccination and screening, we evaluated the prevalence of HR HPV infection and of cervical pre-cancer, and the efficacy of possible screening and treatment strategies, in apparently healthy and previously unscreened Ni-Vanuatu women over 30 years of age.

## Methods

### Subjects and recruitment

A pilot study recruited female subjects aged between 30 and 50 in 2006 by poster and flier advertisement, radio publicity, and nurse “awareness” visits to villages round Port Vila, Efate Island, Vanuatu. Women with a history of gynaecological surgery were excluded. Informed consent was obtained from all participants. A follow on study used a similar recruitment strategy in 2008 amongst non-participants in the first study, and 519 further subjects were recruited. The studies were approved by the Human Experimentation Ethics Committee of the University of Queensland and by the Parliament of the Nation of Vanuatu, and conducted in accordance with the principles expressed in the Declaration of Helsinki. Written informed consent was obtained from all participants. Where necessary this was supplemented by verbal consent in an appropriate language for the participant. The studies are registered in the Australian and New Zealand Clinical Trials Registry (Registration No ACTRN12610000702011).

### Assessment

**Figure 1:**
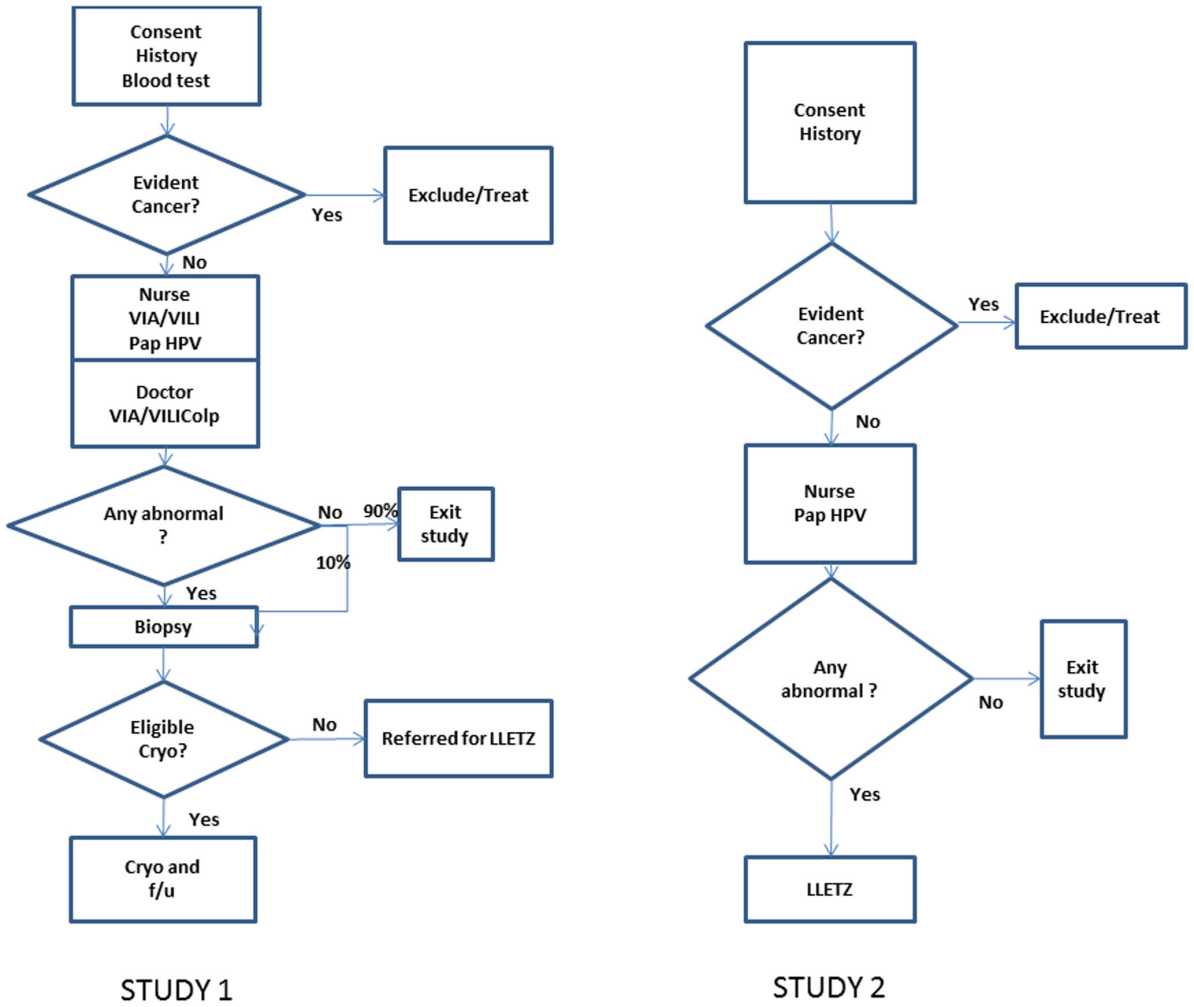
Flow charts for subject recruitment and investigation.

For the pilot study (Figure 1a), a brief medical history and physical examination for hypertension, obesity, visual acuity and diabetes was undertaken. Women underwent a direct light unmagnified speculum examination of the cervix after exposure of the cervix to Acetic Acid (VIA) and again after exposure to Lugols Iodine (VILI). The cervical appearance was reported as within normal limits or significantly abnormal by nursing staff trained and evaluated according to the IARC training manual [11], and, independently, by one of a panel of experienced gynaecologists who were not informed of the findings from the nurse examination. Cervical samples were collected for Pap smear and HPV DNA testing prior to application of Acetic Acid. All women subsequently underwent colposcope aided examination of the cervix by an experienced gynaecologist. Women with any visible abnormality of the cervix or an abnormal pap smear had a colposcopically directed biopsy. Those with local (one or 2 quadrant) cervical disease were then treated by cryotherapy, and those with more extensive disease were referred for loop electrosurgical excision of the cervix transition zone (LLETZ), conducted under local anaesthesia at the Port Vila Hospital. In a second study (Figure 1b), a cervical sample for a Pap smear and HR HPV testing was collected from all recruited subjects. All women testing positive for HR HPV, or with a cytological diagnosis of possible HSIL or worse (Australian classification system), were invited to return for LLETZ.

### Pathology

Cervical cytology samples were taken using a cytobrush and a Cervex sampler, and were processed for both slide and liquid based cytology and for HR HPV DNA testing (Digene HC2 High- Risk HPV DNA Test from QIAGEN). For the first study, samples were also tested for Chlamydia (14 positive of 491 valid samples), for gonorrhoea (2 positive of 492 valid samples) and subjects were also tested for HIV-1 serology (no confirmed positives). Biopsies and LLETZ specimens were fixed in neutral buffered formalin for transportation to Australia. All cytology and histology examinations were conducted by accredited Australian diagnostic pathology providers participating in National Association of Testing Laboratories (NATA) approved quality control and quality assurance schemes for cervical cytology and pathology, and blinded to the clinical and HPV findings. For the pilot study, HPV DNA testing was performed according to the manufacturer's instructions in Australia by a NATA approved laboratory, and for the subsequent study, locally by a trained nurse technician.

#### Statistical Analysis

Statistical analysis was performed using appropriate modules of Statistica V9 (Statsoft, Tulsa, Oklahoma).

## Results

Apparently healthy Ni-Vanuatu women age 30–50 were recruited to a study to evaluate possible strategies for cervical cancer screening. Amongst a study cohort of 499 women (mean age 39.3y ±5.9y), clinically evident cervical cancer was confirmed on biopsy in five subjects who were not included further in the analysis. The remaining women were examined for visual abnormalities of the cervix, by VIA and VILI without magnification, independently by a gynaecologist and a trained nurse, and subsequently colposcopically by a gynaecologist. They were also tested for cytological abnormalities of the cervix using liquid based cytology. If any test were abnormal, subjects underwent colposcopically directed biopsy of the cervix, or LLETZ excision if cancer was suspected clinically. Of 494 subjects evaluated, 206 were biopsied, and 21 were found at biopsy to have CIN 2+.

Correlation between VIA and VILI findings, and between visual inspection findings by doctor and by nurse, was only moderate (Table 1). Amongst 278 subjects with normal visual inspection by all modalities (Table 2), 263 subjects also had normal cervical cytology, of whom a non-random sample of 15 were biopsied with no CIN 2+ detected, and 15 subjects had abnormal cervical cytology (9 HSIL, 6 LSIL) of whom 8 had CIN2+ on biopsy (7 HSIL, 1LSIL). Amongst 214 subjects with abnormal visual inspection by at least one modality, 38 were not biopsied because cytology was normal and at subsequent colposcopy the cervix appeared normal. Of the 176 with abnormal cytology or colposcopic appearance, 12 had biopsy proven CIN 2+. Apparent sensitivity of visual inspection (VILI) for detection of CIN2+ on biopsy was 0.63, specificity was 0.32, and the positive predictive value was 0.09. Overall, cervical cytology was abnormal in 32 of 474 women with technically satisfactory samples (LSIL 13, HSIL 19), and identified 15 of 21 women found to have CIN 2+ on biopsy (1 LSIL, 14 HISL). HSIL cytology had a sensitivity for detection of CIN2+ of 0.99, specificity was 0.77, and the positive predictive value was 0.88.

**Table 1:** 

	Nurse VILI result	
Nurse VIA Result	Normal	Abnormal
**Normal**	318	54
**abnormal**	16	100

	Gynae VILI result	
Gynae VIA Result	Normal	Normal
**Normal**	322	61
**abnormal**	12	89

	Gynae VIA result	
Nurse VIA Result	Normal	Normal
**Normal**	336	35
**abnormal**	52	64

	Gynae VILI result	
Nurse VILI Result	Normal	Normal
**Normal**	295	31
**abnormal**	37	117

**Table 2:** Biopsy findings according to test results at the primary visit in study 1.

		Biopsy findings (positive screen test)			Biopsy findings (negative screen test)			Test Performance (prediction of CIN 2+)@			
Primary screening test		CIN2+	No CIN2+	No Bx*	CIN 2+	No CIN2+	No Bx*	Specificity	Sensitivity	PPV	NPV
**VIA**	**Nurse**	10	92	14	9	84	277	0.48 (0.40–0.55)	0.53 (0.23–0.75)	0.10 (0.05–0.17)	0.90 (0.82–0.95)
	**Doctor**	10	80	9	9	96	282	0.55 (0.47–0.62)	0.53 (0.29–0.75)	0.11 (0.06–0.20)	0.91 (0.84–0.96)
**VILI**	**Nurse**	11	122	20	8	54	270	0.33 (0.26–0.40)	0.58 (0.34–0.79)	0.09 (0.05–0.15)	0.87 (.76–0.94)
	**Doctor**	12	120	15	7	56	271	0.32 (0.25–0.39)	0.63 (0.39–0.83)	0.09 (0.05–0.16)	0.89 (0.78–0.95)
**Colposcopy**		9	23	1	12	150	284	0.87 (0.81–0.91)	0.43 (0.23–0.66)	0.28 (0.14–0.47)	0.93 (0.87–0.96)
**HPV DNA**		14	18	16	7	162	274	0.90 (0.85–0.94)	0.67 (043–0.85)	0.44 (0.27–0.62)	0.96 (0.91–0.98)
**Cytology**	**LSIL+**	16	11	3	5	160	286	0.94 (0.88–0.97)	0.76 (0.52–0.91)	0.59 (0.39–0.77)	0.97 (0.93–0.99)
	**HSIL+**	15	2	2	6	167	287	0.99 (0.95–1.00)	0.71 (0.48–0.88)	0.88 (0.62–0.98)	0.97 (0.92–0.99)

* , If visual inspection was the only abnormal test at the primary visit, and the cervix was visually normal at the biopsy visit, no biopsy was taken.

@ Specificity and PPV for CIN2+: VIA and VILI were significantly less sensitive and less predictive than HPV DNA, LSIL or HSIL (sensitivity p<0.01 in each case; PPV p<0.05 for LSIL, p<0.01 for HSIL). Sensitivity and NPVs were not significantly different between any of the tests.

Most subjects in this study were also tested for high risk (HR) HPV using the liquid based cytology specimen. Of 488 subjects tested for high risk HPV, 44 were positive. A similar rate of positive HPV tests was observed in each 5 year age cohort (30-35y (n = 156), 10.2%; 36-40y (n = 139), 7.9%; 41-45y (n = 99); 12.1%; 46-50y (n = 95), 8.4%). Of the 44 subjects with a positive HPV test, 9 had normal visual inspection and cytology: two of these subjected to biopsy had normal histology. Of the 36 subjects with a positive HPV test and either abnormal cytology or abnormal visual inspection, and therefore subjected to biopsy, 14 had biopsy proven CIN 2+. Normal cytology, if used to triage those with positive HPV tests, would have excluded 12 subjects without CIN 2+ from biopsy, and none with CIN 2+. Of 163 women with normal cytology and a negative HPV test who were subject to biopsy because of abnormal visual inspection, 5 had CIN 2+ on directed biopsy. However, on subsequent LLETZ excision, none had CIN 2+ identified within the pathology specimen.

On specific questioning, a history of post coital or inter-menstrual bleeding was elicited from one of 5 subjects with invasive cancer, none of 21 with biopsy proven CIN 2+, 13 of 182 with normal or low grade pathology on biopsy, and 18 of 291 subjects for whom no biopsy was taken. Thus, elicited symptoms were of no predictive value for selecting women for further screening.

Of 21 subjects from this study with CIN 2+ on biopsy, 15 were predicted by HSIL cytology, of whom 14 also had HR HPV. No subject had CIN2+ predicted only by HR HPV, and the 5 who were detected only by abnormal VIA/VILI were not confirmed on LLETZ excision. Overall, 6 of 21 biopsy identified CIN2+ lesions would have been missed in this study if possible HSIL or worse cytology or a positive test for HR HPV had been used as the screening tests without regard to cervical appearance.

We were aware of the possible ascertainment bias inherent in the first study, in which cytology results, but not HR HPV test results, were used to determine collection of a biopsy in the absence of visual abnormality. The number of women with HR HPV but with normal inspection and cytology, and therefore not subjected to biopsy, and therefore the maximum error in true case ascertainment, was 7, or 25% of subjects with actual or potential CIN2+. We therefore undertook a further study in a new cohort of women drawn from the same population. Women between 30 and 50 (n = 512; Mean age 38.3± SD 5.6) were recruited using the same strategy as for the first study, and tested for abnormal cytology, defined as possible HSIL or worse, and for HR HPV on the same specimen. If either test were abnormal (n = 77), LLETZ excision was offered (Table 3). Of 74 subjects consenting subjects, 27 had CIN 2+ confirmed in the LLETZ specimen. No immediate complications of LLETZ were observed. HSIL cytology (n = 49; mean age 38.3) or a positive HR HPV test (n = 54; Mean age 36.5) were equally sensitive predictors of high grade cervical pathology detected on LLETZ excision, with cytology slightly more specific (Table 4). If abnormality of both tests were set as the screening criterion, the specificity for high grade cervical pathology was better but the sensitivity was significantly less.

**Table 3:** Pathology associated with abnormal HPV test and cytology findings in study 2.

			LLETZ Pathology			
HR HPV	Cytology	Number	CIN2+	CIN1	Normal	Not done
**Negative** **(n = 459)**	**benign**	**359**	0	0	0	359
	**LSIL**	**20**	0	0	0	20
	**HSIL**	**23**	5	7	9	2
	**unsatisfactory**	**57**	0	0	0	57
**Positive** **(n = 53)**	**benign**	**20**	3	10	6	1
	**LSIL**	**5**	1	3	1	0
	**HSIL**	**26**	17	5	3	1
	**unsatisfactory**	**3**	1	0	2	0
**Missing sample** **(n = 7)**	**benign**	**6**	0	0	0	6
	**LSIL**	**0**	0	0	0	0
	**HSIL**	**0**	0	0	0	0
	**unsatisfactory**	**1**	0	0	0	1

**Table 4:** Sensitivity and specificity of HPV testing and Cytology for CIN2+ at LLETZ in study 2.

			LLETZ Findings				Test performance			
Indication for LLETZ	At risk*	Test +ve	No result	CIN 2+	CIN1	Normal	Specificity	Sensitivity	NPV	PPV
**HR HPV+ve or Cyto HSIL**	518	77	4	27	25	21	0.91 (0.88–0.93)	1.00 (0.84–1)	1.00 (0.99–1)	0.37 (0.26–0.49)
**HR HPV +ve**	512	54	2	22	18	12	0.94 (0.91–0.95)	0.81 (0.61–0.93)	0.99 (0.97–1.00)	0.42 (0.29–0.57)
**Cyto HSIL**	458	49	3	22	12	12	0.94 (0.92–0.96)	0.81 (0.61–0.93)	0.99 (0.97–1.00)	0.48 (0.33–0.63)
**HR HPV+ and Cyto HSIL**	453	26	1	17	5	3	0.98 (0.96–0.99)	0.63 (0.42–0.80)	0.98 (0.96–0.99)	0.68 (0.46–0.84)

* At risk subjects were those for whom valid test result(s) were available. There was no significant difference in the performance of HPV testing and cytology for the diagnosis of CIN2+.

## Discussion

In this study we show that cervical cancer is common amongst unscreened apparently healthy Ni-Vanuatu women over 30, with a point prevalence estimate of approximately 100/10^5^ women. Cervical pre-cancer is also common in this population with a point prevalence of about 500/10^5^ women. Biopsy proven CIN 2+ in over 30 year old women is a reliable predictor of lifetime risk of development of cervical cancer [12], as evidenced by its mandatory treatment and follow up in most countries with screening programs, and was therefore used as the gold standard to evaluate possible screening tests in the present study. While histological distinction of CIN2 from CIN3 remains controversial, and CIN2 in younger women is common and of uncertain significance, CIN2 in older women is a marker of HR HPV infection which commonly progresses in this age group to CIN3 [13]. Biopsy diagnosed CIN2 or worse, as determined in an accredited pathology laboratory in Australia, is regarded as of high predictive value for future development of cervical cancer and mandates surgical treatment [14].

To reduce the cervical cancer burden in a low resource setting, a single screening test for women over 30 years of age has been proposed. A single assessment of cervical cytology has a 30%–50% false negative rate for significant cervical pathology, as subsequently detected by colposcopically directed biopsy, as was demonstrated in the current study. Colposcopically directed biopsy can be supplemented by multiple biopsies [15], and is sometimes proposed as a gold standard for assessing cervical pathology in epidemiological studies, but can miss significant pathology [16], particularly within the cervical canal. We chose in the initial study to limit biopsy to women with visual abnormalities or positive cytology, which resulted in 41% of women proceeding to biopsy. We confirmed in a small non-random subset that women with normal visual inspection and normal cytology had a low prevalence of biopsy detected pathology. They also had a low prevalence (7 of 278; 2.5%) of high risk HPV infection, with over 95% sampled for HR HPV. The predictive value of a negative HPV test for absence of pathology amongst women with no cytological abnormalities has recently been reported in a more controlled study (ATHENA) as over 95%. Thus the ascertainment bias in the current study attributable to a policy of not taking a biopsy from the majority of women with normal visual inspection and cytology is likely to be small.

A study of 500 women, even in a country with a high prevalence of disease, has limited power to compare sensitivity and specificity of the various tests. However, visual inspection of the cervix, assisted by acetic acid or Lugols iodine staining, identified 43% of all women in the current study as having significant abnormalities in need of further investigation, a figure significantly higher than the prevalence of disease requiring treatment. The high prevalence of visual abnormalities that would then require treatment, and their relatively poor sensitivity and specificity for high grade premalignant lesions would make visual inspection a poor choice as a feasible single test approach to cervical cancer screening in this population. Thus this study lends support in another geographical setting to the conclusion from a recent report that in India treatment of abnormalities identified through visual inspection is not, in contrast to HPV testing, a practical means of reducing the risk of death from cervical cancer[17].

The high frequency of observed visual abnormality in the current study contrasts with the findings of a meta-analysis of over 52,000 subjects participating in similar studies in which 17% of subjects had abnormal visual inspection. In the meta-analysis, about half the rate of CIN 2+ observed in the current study was reported (2.3% cf 4.2%) [6]. This significant difference may reflect differences in inspection classification, or alternatively a higher prevalence of HPV infection, as suggested by the greater CIN2+ rate, or of intercurrent bacterial and chlamydial infections resulting in abnormality on visual inspection.

The better sensitivity and specificity of cytology for detecting CIN2+ in the current study when compared with the meta-analysis might reasonably be attributed to the use of highly experienced cytology laboratory [18]. In our current study, a significant number of subjects, (7 of 21 in the pilot study and 5 of 27 in the definitive study) had a negative test for HR HPV despite abnormal cytology and biopsy proven CIN 2+. These results are consistent with the meta-analysis, in which HC2 demonstrated a sensitivity of 0.62 and a specificity of 0.93 for detection of CIN2+. However, these figures are at odds with the generally accepted 90% sensitivity of HR HPV testing for biopsy proven CIN 2+ disease in similarly aged cohorts in published studies in the developed world. As the HPV-ve samples that subsequently were shown to come from women with biopsy established cervical pathology in our study demonstrated HSIL on cytology of the same sample, collection of an inadequate sample seems unlikely. Sub-optimal sample handling after collection, under field conditions where samples are exposed to heat and adverse storage conditions and times, might explain loss of HPV assay sensitivity. This needs further evaluation.

Our second study was performed to define the relative utility of abnormal cytology and/or HPV test findings as a screen for CIN 2+, and to establish more precisely the nature of cervical pathology present than was possible by directed biopsy. Relative sensitivity and specificity of HC2 HPV detection for incident CIN 2+ was similar in our two studies, and, consistent with removal of possible ascertainment bias inherent in the first study design, the sensitivity of cytology was somewhat lower.

HC2 HPV detection is somewhat less specific and considerably more sensitive than cytology for detection of CIN2+ lesions in populations preselected by a prior abnormal pap smear[19], and the specificity increases with increasing subject age[20]. The prevalence of high grade lesions amongst those with a positive HR HPV test in the current study approximates 40%. A recommendation was voiced recently for management of patients with a screening test suggestive of cervical pathology (Mark Stoler, International Papillomavirus Workshop, Montreal 2010 – www.hpv2010.org) that where routine colposcopy and biopsy services are available, screening findings with a >40% probability of an underlying high grade cervical lesion should be treated definitively, and those with less should be further assessed. Cryotherapy of visual abnormalities of the cervix has been proposed as a strategy for prevention of progression of CIN2+ to cancer but is not recommended by WHO for large lesions, or lesions within the cervical canal, which are more likely to persist and to progress to cancer [21]–[23] and were common in our current study where 91 of 176 women with visual abnormalities were deemed suitable for cryotherapy, and only 2 of the women suitable for cryotherapy had CIN 2+ on biopsy, representing less than 10% of the subjects with CIN 2+. In a resource poor setting, loop excision of the transformation zone can be safely undertaken in hospital by trained health care practitioners [24], greatly increasing its utility as a cancer control measure. While an optimal screening strategy for cervical cancer prevention might involve primary screening by HPV DNA and subsequent cytology, our data suggest that this strategy would need to involve multiple cytology tests to ensure detection of the majority of CIN2+ lesions associated with a positive HR HPV results. Further, it has been argued that any positive HR HPV test in women over 30y is indicative of a chronic HPV infection, which conveys a significant life time risk of development of cervical cancer even in women without currently apparent cytological abnormalities [25]. Where a cytology triage is not possible, loop excision of the cervix for those testing positive for HR HPV is a feasible strategy for control of cervical cancer, and is likely to remove HPV associated pathology completely in over 90% of patients [9]. The ongoing efficacy of a program based on LLETZ excision of cervical pathology could be evaluated through submission of a random sample of excised material for pathological review in a developed country, to ensure that the expected rate of detection of CIN2+ is being achieved in excised material.

We propose from our findings that a single low cost HPV test, as currently being developed by PATH in cooperation with QIAGEN and the Gates Foundation, with loop excision of the cervix for those with a positive test who have completed their reproductive plan, would be the most appropriate approach to cervical cancer reduction in resource poor countries with high disease prevalence.
